# Supplementary material for: How is enrollees’ trust in health insurers associated with choosing health insurance?
Source: PLoS One. 2023 Nov 2;18(11):e0292964. doi: 10.1371/journal.pone.0292964 (PMC10621964; doi:10.1371/journal.pone.0292964)
Supplement: Attachment — Submitted filename: Response to Reviewers_Trust and HI choice.docx [file pone.0292964.s001.docx]

**Response to Reviewers**

Dear editor,

Thank you for reviewing our paper "What role does enrollees’ trust in health insurers play when choosing health insurance?”, and for giving us the opportunity to react to the issues raised by the academic editor and reviewer. We thank the reviewer for the comments on our manuscript. Based on the comments, we were able to improve our manuscript.

All changes made when revising the manuscript have been highlighted with ‘track changes’ in the revised manuscript. Hereafter, in the rebuttal letter, we provide a point-by-point response to the comments of the academic editor and reviewer.

We hope that the academic editor and reviewer will find our responses to their comments as well as the changes made to the manuscript satisfactory, and that the article is now suitable for publication in PLOS ONE.

Yours sincerely,

On behalf of the authors,

Frank van der Hulst MSc.

**Comments from the academic editor:**

Comment #1: Please ensure that your manuscript meets PLOS ONE's style requirements, including those for file naming. The PLOS ONE style templates can be found at

<https://journals.plos.org/plosone/s/file?id=wjVg/PLOSOne_formatting_sample_main_body.pdf> and 
<https://journals.plos.org/plosone/s/file?id=ba62/PLOSOne_formatting_sample_title_authors_affiliations.pdf>

Reaction to comment #1:

Thank you for addressing PLOS ONE’s style requirements. We have amended the heading levels, table titles, reference citations, acknowledgments section, and the title/authors/affiliations page of the manuscript.

--------------------------------------------------------------------------------------------------------------------------------------

Comment #2: We note that the grant information you provided in the ‘Funding Information’ and ‘Financial Disclosure’ sections do not match.

When you resubmit, please ensure that you provide the correct grant numbers for the awards you received for your study in the ‘Funding Information’ section.

Reaction to comment #2:

A grant number does not apply to this study. However, the information provided in the ‘Funding Information’ and ‘Financial Disclosure’ section need to match. We therefore amended the information provided in the ‘Funding Information’ by adding the Ministry of Health, Welfare and Sport as Funding Source.

--------------------------------------------------------------------------------------------------------------------------------------

Comment #3: Thank you for stating the following financial disclosure:

"The data collection of this study was funded by the Dutch Ministry of Health, Welfare and Sport. The funder had no role in the design, execution and writing of the study."
 
Please state what role the funders took in the study.  If the funders had no role, please state: "The funders had no role in study design, data collection and analysis, decision to publish, or preparation of the manuscript." If this statement is not correct you must amend it as needed.

Please include this amended Role of Funder statement in your cover letter; we will change the online submission form on your behalf.

Reaction to comment #3:

We have amended the Role of Funder statement and added it to our cover letter. Thank you for changing the online submission form on our behalf. The Role of Funder statement has been amended into:

*“The data collection of this study was funded by the Dutch Ministry of Health, Welfare and Sport. The funders had no role in study design, data collection and analysis, decision to publish, or preparation of the manuscript.”*

--------------------------------------------------------------------------------------------------------------------------------------

Comment #4: Thank you for stating the following in the Acknowledgments Section of your manuscript:

"The data collection of this study was funded by the Dutch Ministry of Health, Welfare and Sport. The funder had no role in the design, execution and writing of the study.."

We note that you have provided funding information that is not currently declared in your Funding Statement. However, funding information should not appear in the Acknowledgments section or other areas of your manuscript. We will only publish funding information present in the Funding Statement section of the online submission form.

Please remove any funding-related text from the manuscript and let us know how you would like to update your Funding Statement. Currently, your Funding Statement reads as follows:

"The data collection of this study was funded by the Dutch Ministry of Health, Welfare and Sport. The funder had no role in the design, execution and writing of the study."

Please include your amended statements within your cover letter; we will change the online submission form on your behalf.

Reaction to comment #4:

Thank you for addressing that the funding information should not appear in the Acknowledgements sections or other areas of our manuscript. We have removed it now. As mentioned in the rection to comment #3, we have added the Role of Funder statement to our cover letter, and have amended it into:

*“The data collection of this study was funded by the Dutch Ministry of Health, Welfare and Sport. The funders had no role in study design, data collection and analysis, decision to publish, or preparation of the manuscript.”*

--------------------------------------------------------------------------------------------------------------------------------------

Comment #5: We note that you have indicated that data from this study are available upon request. PLOS only allows data to be available upon request if there are legal or ethical restrictions on sharing data publicly. For more information on unacceptable data access restrictions, please see <http://journals.plos.org/plosone/s/data-availability#loc-unacceptable-data-access-restrictions>.

In your revised cover letter, please address the following prompts:

a) If there are ethical or legal restrictions on sharing a de-identified data set, please explain them in detail (e.g., data contain potentially sensitive information, data are owned by a third-party organization, etc.) and who has imposed them (e.g., an ethics committee). Please also provide contact information for a data access committee, ethics committee, or other institutional body to which data requests may be sent.

b) If there are no restrictions, please upload the minimal anonymized data set necessary to replicate your study findings as either Supporting Information files or to a stable, public repository and provide us with the relevant URLs, DOIs, or accession numbers. For a list of acceptable repositories, please see <http://journals.plos.org/plosone/s/data-availability#loc-recommended-repositories>.

We will update your Data Availability statement on your behalf to reflect the information you provide.

Reaction to comment #5:

We have did now upload the minimal anonymized data set necessary to replicate our study findings to EASY. In the revised version of our cover letter, we explained the data availability as follows:

*“The minimal anonymized data set necessary to replicate our study findings are uploaded to EASY (https://doi.org/10.17026/dans-x5m-hppm). The data set may only be used under the conditions laid down in the privacy regulations of the Dutch Health Care Consumer Panel. Therefore, the dataset is available on request. Data requests will be assessed against these regulations. Additionally, the same minimal anonymized data set is available upon request from prof. Judith D. de Jong (j.dejong@nivel.nl), project leader of the Dutch Health Care Consumer Panel, or the secretary if this panel (conusmentenpanel@nivel.nl). The Dutch Health Care Panel had a program committee, which supervises processing the data of the Dutch Health Care Consumer Panel and decides about the use of the data. This program committee consists of representatives of the Dutch Ministry of Health, Welfare and Sport, the Health Care Inspectorate, Zorgverzekeraars Nederland (Association of Health Care Insurers in the Netherlands), the National Health Care Institute, the Federation of Patients and Consumer Organisations in the Netherlands, the Dutch Healthcare Authority and the Dutch Consumers Association. All research conducted within the Consumer Panel has to be approved by this program committee. The committee assesses whether a specific research fits within the aim of the Consumer Panel, which is to strengthen the position of the health care user.”*

--------------------------------------------------------------------------------------------------------------------------------------

Comment #6: Your ethics statement should only appear in the Methods section of your manuscript. If your ethics statement is written in any section besides the Methods, please delete it from any other section.

Reaction to comment #6:

We have erased the ethics statement from the declaration section (which is now the acknowledgement section) of the manuscript, so this information is now only present in the Materials and Methods section of our manuscript.

--------------------------------------------------------------------------------------------------------------------------------------

**Comments from the reviewers:**

**Reviewer #1**

Comment #1: My largest concern is the panel that responded to the survey seems to be substantially different than the general population. The authors acknowledge this as a limitation from lines 400-408. I would like to know the following things:

1) Is the response rate for this survey similar to other surveys administered to this panel?
2) Is it possible to validate the % of people in restrictive plans with public data. I am unfamiliar with the details of the Dutch health insurance systems but I would imagine market share information should be available.
3) How different is table 3 descriptive statistics for the responding group to the non-responding or general panel group?

I fear that approaches which weigh the data to match population observed characteristics, especially on income and education will still be missing a substantial unobserved characteristic of bureaucratic navigation skill and education. With a voluntary sample that is substantially different from the general panel, I am not sure how to generalize or interpret these results.

Reaction to comment #1:

The reviewer’s concern is that the panel that responded to the survey might be substantially different than the general population. Below, we provide the answers to the three things the reviewer would like to know with respect to this.

1) Yes, the response rate of 56% is similar to other surveys administered to this panel. In general, the response rate of surveys administered to this panel is between 45-60% [1-4]. Literature on Survey research suggests that good survey research has an adequate response rate of at least 40% [5]. Our response rate is higher than [6], or reasonably in line [7] with, average response rates in survey research reported in the literature.
2) As stated in lines 459-461 of the revised manuscript, a comparison of our data with the literature shows that 11% of our respondents reported having a policy with restrictive conditions, which is lower than the national rate of 21.8% reported in the literature [8]. Nevertheless, as we state in lines 461-465, the proportion of young people between aged 18 and 40 years old is underrepresented among our respondents (20% compared to 34% of the national Dutch adult population). As literature shows that most enrollees opting for such a policy are of this age group [9], a deviation between these percentages does not, by definition, mean that there is an insufficient measurement.
3) In the responding group the proportion of young people between aged 18 and 40 years old is underrepresented (20% compared to 34% of the national Dutch adult population) [10]. As we took a sample which is representative regarding gender and age of the adult Dutch population, this means that in the non-respons group younger adults are overrepresented. With regard to gender, the responding group is comparable with the general national Dutch adult population, as well as the total Dutch Health Care Consumer Panel (50% male, 50% female) [10, 11].

With regard to education and health-status, the sample was drawn at random from the total Dutch Health Care Consumer Panel. The health-status of responding group (81% good or very good/excellent) is comparable with the general national Dutch adult population (80,5% good or very good) [12]. As stated in lines 446-447, our respondents are more educated compared to the general national Dutch adult population, thus underrepresenting the less educated [10].

Furthermore, the reviewer indicates to fear that approaches which weigh the data to match population observed characteristics, especially on income and education will still be missing a substantial unobserved characteristic of bureaucratic navigation skill and education. With a voluntary sample that is substantially different from the general panel, the reviewer indicates that he or she is not sure how to generalize or interpret these results.

As we state in lines 446-449, the circumstance that older and more educated enrollees are overrepresented could affect the level of trust we measured. The level of trust we measured is probably higher than actually in the general population, because there were more elderly people in the response group and they might have higher levels of trust [13, 14, 15]. Therefore, following the reviewer's comments, we decided to apply weighting factors by age and gender to the descriptive statistics related to enrollees’ trust in health insurers to correct for this.

To the Materials and Methods section of the manuscript, we added in lines 272-279 the following text with regard to applying weighting factors:

*“Because the survey respondents were not fully representative of all Dutch citizens aged 18 years and older in terms of the combination of age and gender,* *we applied weighting factors by age and gender to the descriptive statistics related to enrollees’ trust in health insurers to correct for this. These weighting factors were calculated by dividing the amount of males and females per age group (18–49, 50–64, and 65+) in the study sample with the corresponding age groups of the Dutch general population as provided by Statistics Netherlands (CBS)[23]. The weighting factors ranged from 0.80 to 1.67 in males, and 0.82 to 1.75 in females.”*

Furthermore, to the Results section, we added the following results in lines 302-303:

*“Corrected with weighting factors by age and gender, these scores are 36.62 and 33.99, respectively (not in table).”*

In addition, in lines 305-306 of the results section, we added the following:

*“When weighting factors by age and gender are applied, these scores are 9.64 and 9.02, respectively (not in table).”*

Furthermore, we added a column with weighted results to Table 4. In relation to that, we added the following to the text of the Results section in lines 317-318:
*“Corrected with weighting factors by age and gender, these percentages are almost identical (Table 4).”*

Lastly, we added in lines 449-451 the following text to the limitations about this:

*“However, the constraint that respondents are relatively older is addressed by the fact that we corrected the results regarding enrollees' trust in health insurers in general with weighting factors by age and gender.”*

We did not apply weighting of these results with regard to income and education. We did not for income, because we do not measured respondents' income in this questionnaire, so we do not have this data. As for education, we made the choice not to correct for this with weight factors, because, apart from applying weight factors for age and gender, we did not want to tinker too much with the results. We have therefore included this point as a limitation in lines 446-449, as mentioned earlier.

Furthermore, as stated in lines 451-452 our regression results are not expected to have been affected by the circumstance that older and more educated enrollees are overrepresented, as all subgroups are sufficiently large to perform association analyses. Inclusion of the background variables in the logistic regression models, including age and education, ensures that there is control for confounders.

References:

1. Holst, L., Victoor, A., Brabers, A., Rademakers, J., & de Jong, J. (2022). Health insurance literacy in the Netherlands: The translation and validation of the United States’ Health Insurance Literacy Measure (HILM). Plos one, 17(9), e0273996.
2. van der Hulst, F. J., Brabers, A. E., & de Jong, J. D. (2023). The relation between trust and the willingness of enrollees to receive healthcare advice from their health insurer. BMC Health Services Research, 23(1), 1-11.
3. van der Hulst, F. J., Holst, L., Brabers, A. E., & de Jong, J. D. (2022). To what degree are health insurance enrollees in the Netherlands aware of the restrictive conditions attached to their policies?. Health Policy, 126(7), 693-703.X
4. Brabers, A. E., Rademakers, J. J., Groenewegen, P. P., Van Dijk, L., & De Jong, J. D. (2017). What role does health literacy play in patients' involvement in medical decision-making?. PloS one, 12(3), e0173316.
5. Story, D. A., & Tait, A. R. (2019). Survey research. Anesthesiology, 130(2), 192-202.
6. Wu, M. J., Zhao, K., & Fils-Aime, F. (2022). Response rates of online surveys in published research: A meta-analysis. Computers in Human Behavior Reports, 100206.
7. Holtom, B., Baruch, Y., Aguinis, H., & A Ballinger, G. (2022). Survey response rates: Trends and a validity assessment framework. human relations, 00187267211070769.
8. NZa. Monitor zorgverzekeringsmarkt 2022. Utrecht: NZa; 2022 1 November 2022.
9. NZa. Polissen met beperkt aantal contracten in de medisch specialistische zorg. Analyse van aantal, aard en kenmerken van deze polissen, in opdracht van het ministerie van VWS. Utrecht: NZa; 2020 8 October 2020.
10. Bevolking op 1 januari en gemiddeld; geslacht, leeftijd en regio. In: CBS, editor. 2022.
11. Brabers A, de Jong J. Nivel Consumentenpanel Gezondheidszorg: basisrapport met informatie over het panel 2022 [Nivel Health Care Consumer Panel: Basic report with information on the panel 2022]. 2022.
12. CBS. Monitor Brede Welvaart & Sustainable Development Goals 2022. Gezondheid [cited 2023 12 June]; Available from: <https://www.cbs.nl/nl-nl/dossier/brede-welvaart-en-de-sustainable-development-goals/monitor-brede-welvaart-sustainable-development-goals-2022/verdeling/indicatoren/gezondheid#1>
13. Hall, M.A., et al., Trust in physicians and medical institutions: what is it, can it be measured, and does it matter? The milbank quarterly, 2001. 79(4): p. 613-639.
14. Li, T. and H.H. Fung, Age differences in trust: An investigation across 38 countries. Journals of Gerontology Series B: Psychological Sciences and Social Sciences, 2013. 68(3): p. 347-355.
15. Bailey, P.E. and T. Leon, A systematic review and meta-analysis of age-related differences in trust. Psychology and aging, 2019. 34(5): p. 674.

--------------------------------------------------------------------------------------------------------------------------------------

Comment #2: Switching behaviors --- what is the look back period for the switch insurance? I don't know if it is in the past contract period or ever.

Reaction to comment #2: In the Netherlands enrollees can switch health insurance or change their policies from November 12. The last day to decide to discontinue a health insurance policy is December 31. The last day to enroll in a new health insurance is January 31. The new health insurance then starts January 1, with retroactive effect if enrolled to a new health insurance policy in January. As the survey was send out in February 2022, we examined their switching behaviour between November 12, 2021, and January 31, 2022, for the contract period of 2022.

In lines 74-79 we added the following text to the manuscript:

*“Every year, from November 12, enrollees have the option of switching to another health insurer or health insurance policy for the coming year. The last day to enroll in a new health insurance is January 31. The new health insurance then starts January 1, retroactively if enrollees enroll in a new health insurance policy in January.”*

--------------------------------------------------------------------------------------------------------------------------------------

Comment #3: Table 7 -- I am unsure how to interpret AGE -- do older people have higher or lower odds of switching? I can't tell direction here. Please clarify.

Reaction to comment #3:

We stated that younger enrollees have switched health insurers slightly more often (OR=0.94, p=0.00). We understand that this may be somewhat confusing, since the OR is related to age in a direction that is increasing. The OR of 0.94 means that older people have a lower odds of switching compared to younger people. Therefore, we have amended this statement on younger enrollees to a statement on older enrollees. The text in lines 338-340 now reads:

*“If we look at the background characteristics, older enrollees have switched health insurers slightly less often than younger enrollees (OR=0.94, p<0.001).”*

--------------------------------------------------------------------------------------------------------------------------------------

Comment #4: P=0.00 makes no sense to me, is it better to say p<.01 or p<.0001 as there is some miniscule chance of a random draw from a distribution centered on the null to produce a result as unusual as the ones that are observed.

Reaction to comment #4:

Thank you for addressing this point. Where applicable, we have changed p=0.00 to p<0.01 or p<0.001 in the manuscript.

--------------------------------------------------------------------------------------------------------------------------------------

Comment #5: Table 8 hints that there is a rational response to more restrictive insurance by individuals who are less likely to use insurance or who are severely cash-constrained. Is it possible to incorporate income into these analyses to further examine the role of resource availability and choice?

Reaction to comment #5:

We think this is an interesting point, but unfortunately we do not have information on respondents' income. However, it is a good suggestion to include in follow-up research.

--------------------------------------------------------------------------------------------------------------------------------------
