# Supplementary material for: How is enrollees’ trust in health insurers associated with choosing health insurance?
Source: PLoS One. 2023 Nov 2;18(11):e0292964. doi: 10.1371/journal.pone.0292964 (PMC10621964; doi:10.1371/journal.pone.0292964)
Supplement: Attachment — Submitted filename: Response to Reviewers_Trust and HI choice.docx [file pone.0292964.s002.docx]

**Response to Reviewers**

Dear editor,

Thank you for reviewing our paper "What role does enrollees’ trust in health insurers play when choosing health insurance?”, and for giving us the opportunity to react to the points raised by the reviewer. We thank the reviewer for the comments on our manuscript. Based on the comments, we were able to improve our manuscript.

All changes made when revising the manuscript have been highlighted with ‘track changes’ in the revised manuscript. Hereafter, in the rebuttal letter, we provide a point-by-point response to the comments of the reviewer.

We hope that the reviewer will find our responses to the comments as well as the changes made to the manuscript satisfactory, and that the article is now suitable for publication in PLOS ONE.

Yours sincerely,

On behalf of the authors,

Frank van der Hulst MSc.

**Comments from the reviewers:**

**Reviewer #1**

Comment #1: I would like a title change to indicate an association. The analysis is, in my opinion, non-causal. It is a very useful descriptive piece that highlights associations of trust and health insurance selection but I am not seeing an RCT or the standard set of quasi-experimental designs (DiD, ITS, regression discontinuities etc). This is recognized in line 452.

Reaction to comment #1: The reviewer is right that the analysis may indicate association rather than causation. We therefore agree with the reviewer's request to change the title. We have amended the title of this paper into:

*“How is enrollees' trust in health insurers associated with choosing health insurance?”*

Furthermore, we noticed that at every point in the manuscript where we described the aim of the study, it was written, as in the title, as examining causation rather than association. We therefore made some additional changes. For example, line 6 described: “This study aims to investigate what role enrollees’ trust in health insurers plays in their choice of a health insurance policy in the Netherlands”. We changed this into:

*“This study aims to investigate how enrollees’ trust in health insurers is associated with their choice of a health insurance policy in the Netherlands.”*

--------------------------------------------------------------------------------------------------------------------------------------

Comment #2: Line 79 “There is a lot to choose from....” Is either not needed or needs to be clarified what THERE refers to.

Reaction to comment #2: We agree with the reviewer that this short sentence is unclear and does not add value. We have therefore decided to omit this sentence.

--------------------------------------------------------------------------------------------------------------------------------------

Comment #3: Table 8, please include a precis of each model in the line that includes the model number and N.

Reaction to comment #3: The reviewer is right that it would be clearer to include a precis of each model in Table 8. We have therefore slightly modified the main title of Table 8 and added a precis to each model in the table. For model 2, this is "*Examining the association with general trust in health insurers"*, for model 3, "*Examining the association with trust in one's own health insurer*" and for model 4, "*Examining the association with trust in one's own health insurer's purchasing strategy.*”
